# The functional role of p38 MAPK pathway in malignant brain tumors

**DOI:** 10.3389/fphar.2022.975197

**Published:** 2022-10-03

**Authors:** Nathália Grave, Thamiris Becker Scheffel, Fernanda Fernandes Cruz, Liliana Rockenbach, Márcia Inês Goettert, Stefan Laufer, Fernanda Bueno Morrone

**Affiliations:** ^1^ Programa de Pós-Graduação em Medicina e Ciências da Saúde, Escola de Medicina, Pontifícia Universidade Católica do Rio Grande do Sul, Porto Alegre, Rio Grande do Sul, Brazil; ^2^ Laboratório de Farmacologia Aplicada, Escola de Ciências da Saúde e da Vida, Pontifícia Universidade Católica do Rio Grande do Sul, Porto Alegre, Rio Grande do Sul, Brazil; ^3^ Programa de Pós-Graduação em Biologia Celular e Molecular, Escola de Ciências da Saúde e da Vida, Pontifícia Universidade Católica do Rio Grande do Sul, Porto Alegre, Rio Grande do Sul, Brazil; ^4^ Laboratorio de Cultura de Células, Programa de Pós-Graduação em Biotecnologia, Universidade do Vale do Taquari (Univates), Lajeado, Brazil; ^5^ Department of Pharmaceutical and Medicinal Chemistry, Institute of Pharmacy, Eberhard Karls University of Tübingen, Tübingen, Germany

**Keywords:** glioma, mitogen-activated protein kinases, p38 MAPK inhibitors, small molecules, brain tumors

## Abstract

Gliomas are extremely debilitating malignant brain tumors with very limited response to therapies. The initiation and progression of gliomas can be attributed to several molecular abnormalities, such as mutations in important regulatory networks. In this regard, the mitogen-activated protein kinases (MAPKs) arise as key signaling pathways involved in cell proliferation, survival, and differentiation. MAPK pathway has been altered in most glial tumors. In glioma cells, the activation of p38 MAPK contributes to tumor invasion and metastasis and is positively correlated with tumor grade, being considered a potential oncogenic factor contributing to brain tumorigenesis and chemotherapy resistance. Hence, a better understanding of glioma pathogenesis is essential to the advancement of therapies that provide extended life expectancy for glioma patients. This review aims to explore the role of the p38 MAPK pathway in the genesis and progression of malignant brain tumors.

## Introduction

Malignant brain tumors are rapidly growing cancer with high invasion potential to surrounding healthy brain structures, which include gliomas and neuronal or mixed neuronal-glial tumors (Lapointe et al., 2018). Gliomas are the most common central nervous system (CNS) cancers, comprising approximately 80% of all brain malignancies in adults (Hanif et al., 2017; Morrone et al., 2021). The heterogeneity and high invasiveness are hallmarks implicated in the poor prognosis of these tumors (Colquhoun, 2017; Reni et al., 2017).

The initiation and progression of gliomas can be attributed to several molecular abnormalities, such as mutations in important regulatory networks (Morrone et al., 2016; Pandey et al., 2016). The mitogen-activated protein kinases (MAPKs) are key signaling pathways involved in the regulation of cell proliferation, survival, and differentiation (Gao et al., 2005). In this regard, the MAPK pathway has been widely studied and is altered in most glial tumors. In gliomas, this pathway has been associated with poor prognosis, and resistance to radiotherapy, and appears to regulate, directly and indirectly, its genesis and progression through crucial signaling mediators (Patil et al., 2013; Pandey et al., 2016).

Among the MAPK pathways, p38 mitogen-activated protein kinase (p38 MAPK) signaling was identified as a mediator of stress and inflammation responses (Ono and Han, 2000). This pathway is involved in the biology of different types of cancer, and plays an extremely significant role in cancer progression, such as proliferation, invasion, and survival, and has been widely studied as a potential pharmacological target (Wagner and Nebreda, 2009; Roche et al., 2020). Originally described as a tumor suppressor kinase, p38 has a dual role in tumorigenesis, also acting as a tumor promoter (Koul et al., 2013; Martínez-Limón et al., 2020). In gliomas, phosphorylated p38 MAPK has been considered a potential biomarker of progression once its activation contributes to tumor invasion and metastasis and is positively correlated with the tumor grade (Glassmann et al., 2011; Pandey et al., 2016).

Considering the importance of the signaling pathways involved in the genetic and molecular alterations in the onset of cancer, describing the role of the p38 MAPK pathway in tumor microenvironment and in the progression of gliomas becomes a key strategy to comprehend and propose new treatments for malignant brain tumors.

## The glioma microenvironment

Malignant brain tumors are aggressive and impact directly patients’ life quality (Omuro, 2013). Gliomas are representative of around 25% of CNS cancers and are classified according to their similarities with glial cells from which arise, and astrocytomas are the most frequent type. Their classification includes both molecular and histological parameters and encompasses two principal subgroups: diffuse and non-diffuse gliomas. Diffuse gliomas reveal a massive infiltrative growth in the surrounding parenchyma and are more genetically heterogenic in their composition and difficult to treat when compared to non-diffuse gliomas (Omuro, 2013; Perry and Wesseling, 2016; Ostrom et al., 2018).

Glioblastoma (GBM) is the highest grade of diffuse astrocytoma and is considered incurable (DeAngelis, 2001; Omuro, 2013; Perry and Wesseling, 2016; Ostrom et al., 2018). Despite the improvements in therapeutic research, maximal surgical resection followed by radiotherapy and chemotherapy remains the main standard of treatment. Tumor recurrence and therapeutic resistance are frequent, so the median survival rate is around 15 months. Hence, treatments of malignant brain tumors remain challenging (Lin et al., 2017).

It is well known that molecular characteristics inside the tumor microenvironment (TME) indicate the sensitivity of tumors to therapy. Initially, tumorigenesis is related to the gain of numerous genetic mutations, which provide malignant cells resistant to the growth-inhibitory and apoptotic signals (Hanahan and Weinberg, 2011; Kelly and Strasser, 2011). All GBM have common oncogenes likely to carry a growth advantage, which are considered driver mutations for their pathogenesis. As pointed in the literature, well characterized oncogenes and tumor suppressor genes perform critical roles in several signaling pathways, being great influencers in biological behaviors and in glioma heterogeneity (Eder and Kalman, 2014).

In general, tumors are complex tissues composed of distinct cell types that interact with each other forming an intense signaling network (Hanahan and Weinberg, 2011). The glioma microenvironment includes non-neoplastic cells, GBM stem cells (GSCs), fibroblasts, immune cells, tumor-associated macrophages (TAMs), endothelial cells, and vascular pericytes. In addition, TME also includes proteins and non-protein biomolecules produced by all of these distinct cell types, supporting the various processes of tumor promotion (Schiffer et al., 2018). Numerous interrelated pathways are frequently mutated in the glioma microenvironment, typically those controlling cell-cycle, cellular survival, invasion, and angiogenesis. These alterations result in tumor self-sufficiency with growth signals, cell death resistance, growth suppressors evasion, sustained angiogenesis, and tissue invasion (Pojo and Marques, 2011; Nørøxe et al., 2017).

Hypoxia is considered a hallmark of GBM, and a critical factor associated with mutations in multiple signaling pathways that affect the tumor landscape (Oliver et al., 2009; Schiffer et al., 2018). Solid tumors grow fast, surpassing vascular supply and consequently, inhibiting oxygen diffusion. Several hypoxia-mediated signaling pathways play a role in angiogenesis, tumor proliferation, and immunosuppression (Mohan et al., 2021; Scheffel et al., 2021). Glioma cells, under hypoxic conditions, secrete interleukins and chemokines, which provide proliferative signals and have been implicated in the stimulation of angiogenesis. Likewise, extracellular adenosine increases in hypoxic conditions and is associated with tumor cell proliferation through a variety of pathways including MAPK signaling and the upregulation of cyclin proteins (Zhou et al., 2017; Scheffel et al., 2021).

## The mitogen-activated protein kinases pathway

Protein kinases are enzymes that have the property to catalyze protein phosphorylation through the transfer of a phosphoryl group from ATP and GTP to threonine, serine (Ser-/Thr-specific protein kinases; S/T PKs), tyrosine (Tyr- specific protein kinases; T PKs), both serine/threonine and tyrosine (dual-specificity protein kinases) (Cohen, 2002; Baier and Szyszka, 2020). The phosphorylation of these amino acids residues triggers extra and intracellular stimuli, orchestrating a highly efficient mechanism for the control of protein activity (Bononi et al., 2011). About 30% of all human proteins can be altered by kinases activity, which can regulate most cellular pathways, particularly those related to signal transduction (Ardito et al., 2017).

MAPKs are Ser/Thr kinases ubiquitously expressed, that regulate cellular mechanisms in response to a wide range of stimuli including cytokines, growth factors, antigens, toxins, drugs, osmolarity, temperature, oxygen radicals, ultraviolet light, changes in cell shape, adherence, and cell-cell interactions (Pearson et al., 2001; Cuevas et al., 2007; Lee et al., 2020). In mammals, 14 MAPKs have been described so far, characterized in seven groups. The conventional MAPKs include the extracellular signal-regulated kinases 1/2 (ERK1/2), c-Jun amino (N)-terminal kinases 1/2/3 (JNK1/2/3), p38 isoforms (*α*, *β*, *γ*, and *δ*), and ERK5. Atypical MAPKs have nonconforming particularities and consist of ERK3/4, ERK7, and Nemo-like kinase (NLK) (Cargnello and Roux, 2011).

The activation of MAPKs and their downstream targets plays a pivotal role in different signaling cascades involved in transcription, development, differentiation, migration, cell death, and many other critical cellular functions (Geest and Coffer, 2009; Yue and Lopez, 2020). Detriment in MAPKs signaling due to mutation or altered expression of proteins regulating MAPKs cascades can lead to pathological conditions such as cancer, neurodegeneration, inflammation, and developmental defects (Johnson et al., 2005; Kim and Choi, 2015).

MAPKs signaling cascades present recurrent contributions to oncogenesis, tumor progression, and drug resistance, which suggests that its pharmacological modulation can be a promising strategy in the development of cancer therapies (Chaikuad et al., 2018; Braicu et al., 2019; Wittlinger and Laufer, 2021). It is well known that MAPKs can regulate cellular events directly related to tumor development, such as proliferation, apoptosis, inflammation, and immunity (Kim and Choi, 2015). JNK, ERK1/2, and p38 have been described as the most involved in the carcinogenesis processes (Sebolt-Leopold and Herrera, 2004; Lei et al., 2014; Kim and Choi, 2015; Peluso et al., 2019).

### The p38 MAPK pathway and cancer

Discovered on a pharmacological screen in 1994, p38 MAPK is a signal transduction pathway that plays a key role in cellular adaptation to extracellular stimuli (Han et al., 1994; Yong et al., 2009; Zou and Blank, 2017). Its activation is often in response to various environmental and cellular stresses, inflammation, and other signals such as UV irradiation, oxidative stress, and exposure to DNA damaging agents, as well as growth factors and cytokines (Loesch and Chen, 2008; Keshet and Seger, 2010).

After extracellular signals, activation of the p38 pathway usually proceeds through a classical phosphorylation cascade, where a MAPKKK (ASK1, TPL2, MEKK3) phosphorylates and activates the MAPKKs specifics of p38 MAPK, MKK3, and 6, which in turn, mediate the activation of the different p38 MAPK isoforms (Cuadrado and Nebreda, 2010; Stramucci et al., 2018). The p38 sometimes can also be phosphorylated by MKK4, a kinase well known as a JNK activator (Brancho et al., 2003). Once activated, p38 can phosphorylate many cytosolic proteins as Bcl-2 family and tau proteins. Besides the phosphorylation of cytosolic proteins, p38 can be translocated from the cytosol to the nucleus, where it phosphorylates, among other, the MSK 1 and 2, which in turn can phosphorylate histone H3 and the related transcription factors CREB and ATF1. This p38 phosphorylation cascade regulates cellular responses ranging from apoptosis to cell division, cell invasion, and inflammatory response (Deacon et al., 2003; Cuadrado and Nebreda, 2010; Reyskens and Arthur, 2016).

The p38 MAPK family compromises four isoforms: p38α (also known as MAPK14 or SAPK2a), p38β (MAPK11, SAPK2b), p38γ (MAPK12, SAPK3, ERK6), and p38δ (MAPK13, SAPK4) (Zou and Blank, 2017). The four isoforms share more than 60% homology and are widely expressed; however, each isoform has different tissue-specific expression patterns (Cuenda and Sanz-Ezquerro, 2017; Mai et al., 2020). The isoform p38α is expressed in most cell types and is widely cited (Wagner and Nebreda, 2009). In contrast, p38β is expressed at very low levels, being restricted to the brain and lung. The p38γ and p38δ have more limited expression and are likely to have specialized functions. While p38γ is mostly detected in skeletal muscle and nervous system, the p38δ is enriched in uterus and pancreas (Ono and Han, 2000; Cuenda and Rousseau, 2007).

In recent years, the involvement of p38 MAPK in cancer has been widely described (Zou and Blank, 2017; Martínez-Limón et al., 2020; Lee et al., 2020; Anton et al., 2021). This pathway, as well as several other signaling cascades, regulates the balance between cell survival and death in response to stress, which impacts directly on tumorigenesis (Hanahan and Weinberg, 2011; Grossi et al., 2014). This regulation, depending on the type and strength of stress, cell type, and the interference among other signaling pathways can lead to opposite cell fates of survival or death (Dhillon et al., 2007; Wagner and Nebreda, 2009).

Some studies have suggested a role for p38 MAPK in mediating pathways that lead to cell apoptosis and growth inhibitory signals, particularly in promoting cell cycle arrest and differentiation, which supports the idea that the stress-activated kinase is a tumor suppressor (Bulavin and Fornace, 2004; Olson and Hallahan, 2004; Koul et al., 2013). However, other studies have shown that activation of this signaling pathway can produce exactly the opposite effect such as anti-apoptotic and proliferative effects, enhancing cell survival pathways, migration, or resistance to stress and chemotherapeutic agents in tumor cells (Wada and Penninger, 2004; Wagner and Nebreda, 2009; Martínez-Limón et al., 2020). The p38 MAPK has also been related to cell death by apoptosis, which led us to consider this pathway as a key factor in the response to chemotherapy (O’Callaghan et al., 2015; García-Cano et al., 2016).

### Malignant brain tumors and p38 MAPK signaling

MAPKs are expressed in various types of malignant brain tumors (Jones, et al., 2012; Soeda et al., 2017). Besides, p38 is upregulated GBM cell lines as well as in GBM patients (Demuth et al., 2007; Pandey et al., 2016). This protein is shown to be involved in the response of diverse molecules, it is mainly implicated in inflammation, proliferation, migration, invasion, and ROS signaling been involved in GBM initiation, progression, metastasis, and chemotherapy response (Yeung et al., 2013; Pandey et al., 2016).

Among diverse brain tumor types, glioma presents one of the worst patients’ prognostics. GBMs are hard to treat and avoid recurrence due to its infiltrative pattern. The involvement of p38 MAPK in the GBM migration and invasion has been extensively described (Park et al., 2006; Yoshino et al., 2006; Demuth et al., 2007; Chen et al., 2020). Demuth et al. (2007) have identified the MKK3 as a key activator of GBM invasiveness through p38 activation, both *in vitro* and *in vivo*. Irradiation is often part of the GBM treatment scheme, although, in cells that present mutant PTEN it was described that irradiation can activate p38/Akt and PI3K/Akt signaling pathways, increasing MMP-2 expression and intensifying invasiveness (Park et al., 2006).

Long noncoding RNAs (lncRNAs) are transcripts with more than 200 nucleotides that commonly do not encode proteins, many of them have been characterized as oncogenes or tumor suppressors in cancer (Ma et al., 2018). The MRCCAT1 is an oncogenic lncRNA that promotes proliferation and migration of GBM cells *via* p38 MAPK signaling activation. Moreover, lncRNA-ATB promotes invasiveness mediated by tumor growth factor-β (TGF-β) which activates the p38 MAPK pathway (Tang et al., 2019).

Nowadays it is known that, in tumor progression, gliomas orchestrate the immune system to a protumoral phenotype. The p38 MAPK is also correlated with immune and inflammation signaling, being responsible for the production of cytokines in the TME (Choi et al., 2001; Schieven, 2009; Kühnöl et al., 2013; Wang et al., 2019). Wang et al. (2019) have been shown that MXB (also named MX2) protein, a component of the innate immune response, is downregulated in GBM cells. This fact corroborates to tumor progression once MXB overexpression decrease the cell proliferation, invasion, and migration due to the decrease in ERK1/2 and p38 phosphorylation/activation, and Nf-κB levels (Wang et al., 2019).

**FIGURE 1:**
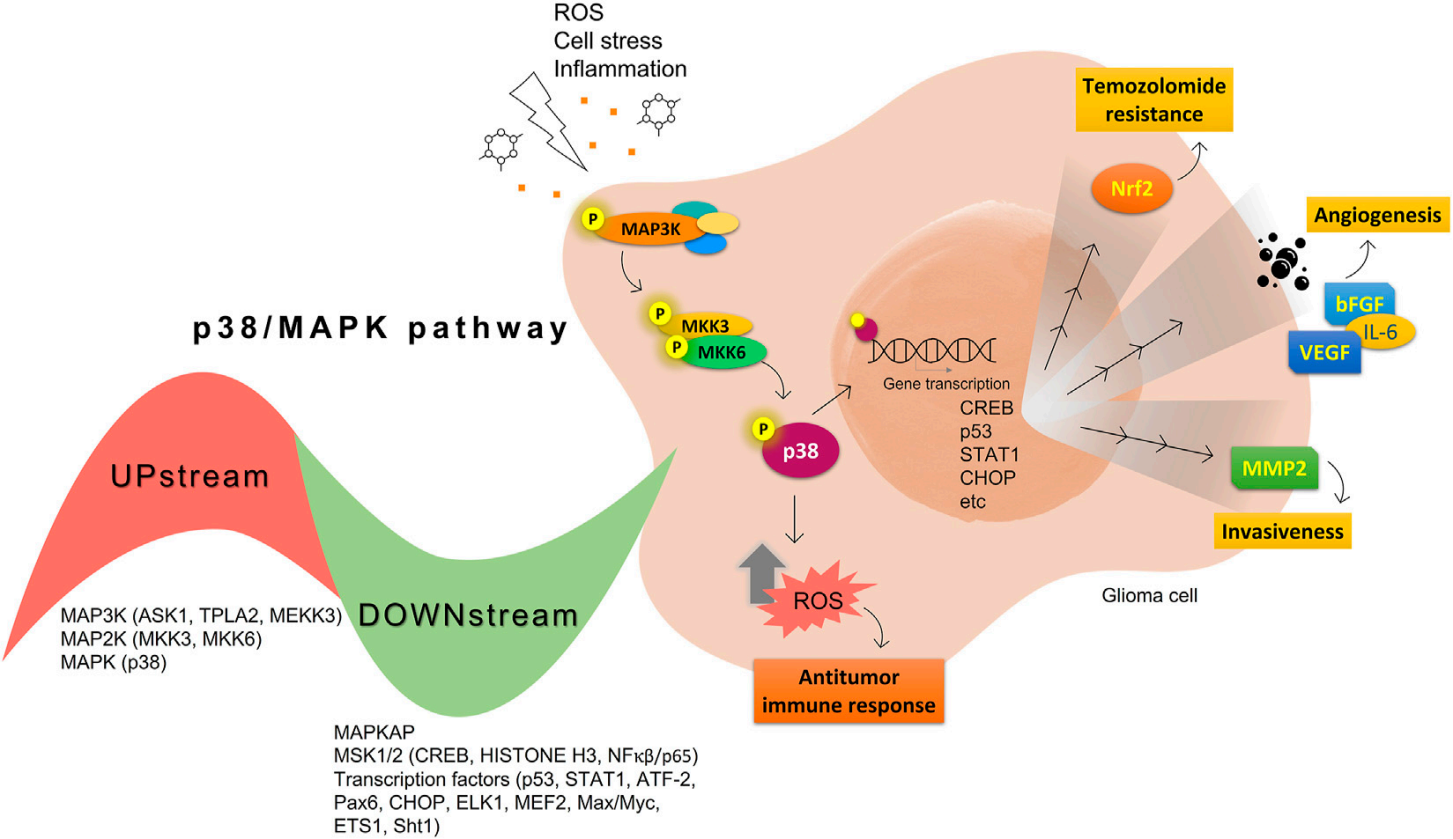
Summary of protumoral (yellow) and antitumoral (orange) p38 MAPK activation effects on glioma cells.

In this context, it is well known that angiogenesis is essential for tumor progression, T98G, U373, and U87 glioma cell lines have been revealed the p38 MAPK and JNK constitutively activated contributing angiogenesis induced by the vascular endothelial growth factor (VEGF) release (Yoshino et al., 2006). Other studies have shown that MAPKs and hypoxia can control VEGF expression in cell model (Berra et al., 2000). Besides, VEGF, p38 still contributes to the basic fibroblast growth factor (bFGF), epidermal growth factor (EGF), IL-6, and another proangiogenic cytokine secretion (Tate et al., 2013). The p38 MAPK inhibition on glioma cells showed decreased VEGF secretion (Yoshino et al., 2006) and enhanced immune responses *in vitro* (Kühnöl et al., 2013). Figure 1 summarizes the p38 activation effects on glioma cells.

### The p38 MAPK inhibitors in gliomas

Several small-molecule protein kinase inhibitors have been tested for cancer treatment, demonstrating interesting effects on the immune system. Among these, there is the EGFR tyrosine kinase inhibitor gefitinib, and other inhibitors focusing on multiple targets, like imatinib, sorafenib, and sunitinib (Ott and Adams, 2011). Regardless of p38 MAPK dual role as a possible oncogene or tumor suppressor, this signaling pathway has attracted much attention as a promising drug target for cancer therapy based on small inhibitory compounds (Borst et al., 2013; Koul et al., 2013; Haller et al., 2020). Since its first inhibitor, a pyridinylimidazole compound, described in 1994, numerous inhibitors against p38 MAPKs have been reported with diverse chemical structures (Han et al., 1994; Zhang et al., 2007; Yong et al., 2009; Borst et al., 2013; Haller et al., 2020). These inhibitors are divided into two groups, depending on their mode of binding: the direct inhibitors, such as SB203580, and the majority of the p38 inhibitors, which binds competitively to the ATP-binding site; and the indirect inhibitors such as BIRB-796, which inhibits p38 MAPK activity by conformational change (Zhang et al., 2007; Yong et al., 2009; Haller et al., 2020). Regarding GBM proliferation, a study has shown that the p38γ is correlated with the grade of glioma malignancy and promotes proliferation and progression (Yang et al., 2013). Cycloartenal is an important triterpenoid prevalently found in plants. This compound inhibits p38 MAPK activation and demonstrated antiproliferative effects in U87 human glioma cells (Niu et al., 2018).

Once p38 MAPK is involved in glioma proliferation, researchers have investigated the potential of p38 MAPK inhibitors in inducing cell death in GBM by itself or in association with other treatments (Table 1). The LY479754 p38 inhibitor increased temozolomide anti-glioma effects in U87 and SNB19 spheroids when used in combination for 5 days (Demuth et al., 2007). The SB203580 p38 MAPK inhibitor potentialized the antiproliferative effects of temozolomide when U87 and U251 cells were treated for 24 h *via* Nrf2 signaling inhibition, indicating that p38 MAPK/Nrf2 activation is a network involved in temozolomide glioma cells resistance (Ma et al., 2015). Curiously, a study has shown that the circadian clock has an important role in the activation of the p38 MAPK pathway, which means that the treatment with p38 MAPK inhibitors may be more effective and less toxic depending on the time the therapy is administered (Goldsmith et al., 2018).

**TABLE 1:** Use of p38 MAPK inhibitors in pre-clinical studies on glioma cell lines.

Agent/p38 MAPK inhibitor structure	Cell line	Outcome	Reference
**BIRB796**	U87	Autophagy reduction: lower fluorescence intensity and the lower number of autophagic vacuoles per cell treated.	Xu et al. (2020)
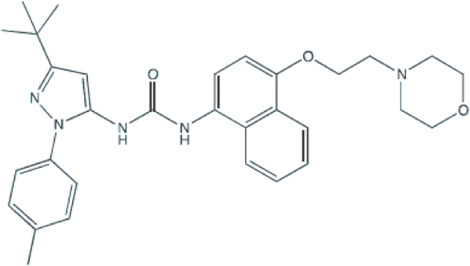	U87 e U251	Blocked the G1 phase cell cycle and decreased S and G2 phases; And inhibited the proliferation, migration, and invasion of GBM cell lines.	Zhao et al. (2021)
**SB203580** 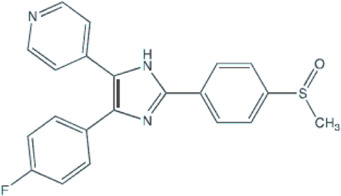	F98	Decreased ROS production and the cell number in early apoptosis.	Li et al. (2018)
SB203580 (and TMZ)	U87 and U251	Increased the sensitivity of glioma cells to TMZ.	Ma et al. (2015)
SB203580 (plus Anisomycin and TMZ)	U87 and U251	SB203580 reduced anisomycin and TMZ antitumoral effects promoting migration and invasion.	Chen et al. (2017)
SB203580 (and Vandetanib)	SF767 and U251	Synergism on antitumoral effect against glioblastoma cells.	Sooman et al. (2013)
SB203580 (and Anisomycin)	GBM: X01, X02, X04, X05, X06, 08–322, 08–387; X07 Gliosarcoma; and X03Anaplastic oligoastrocytoma	Decreased proliferation. Increased the undifferentiated GSC population and apoptotic events.	Soeda et al. (2017)
SB203580 (plus Arenobufagin and Hellebrigenin)	U87 and primary mouse glial cells	SB203580 decreased cell viability and enhanced the cytotoxicity of arenobufagin and of hellebrigenin.	Han et al. (2018)
LY2228820/Ralimetinibe (and PD-L1 antibody) 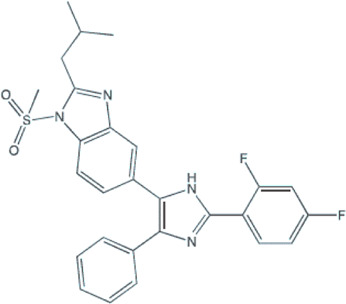	GL261-C57BL/6 mice	Enhanced survival of mice with temozolomide-resistant glioma-bearing with reduction of the accumulation of macrophages/microglia.	Dang et al. (2021)
**069A** 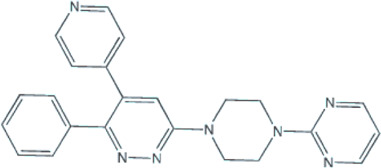	U251	Inhibited migration, invasion and the antitumoral inflammatory response.	Yeung et al. (2012)

Abbreviations: GBM, glioblastoma; MK2, kinase MAPK-activated protein kinase 2; TMZ, temozolomide.

Glioma cells undergo metabolism reprogramming due to high proliferation rates and elevated consumption of glucose in this process. TGF-β participates in this mechanism, and an interesting study showed that TGF-β activated PFKFB3 expression and activity *via* activation of the transcript factor Smad, p38 MAPK e PI3K/Akt signaling pathways while p38 MAPK inhibition reversed TGF-β stimulatory effects (Rodríguez-García et al., 2017).

Since p38 is a ubiquitous protein, treatment of gliomas with p38 MAPK inhibitors may be successful, however, the involvement of this pathway in multiple cellular processes may result in ambiguous effects. An interesting study showed that temozolomide inhibited proliferation, migration, and invasion in U87 and U251 cell lines, and these effects were associated with a decrease in the levels of AQP4 dependent on p38 MAPK activation. When the p38 MAPK inhibitor (SB203580) was used in combination with temozolomide, it reduced temozolomide antitumoral effects (Chen et al., 2017). Besides, treatment with SB203580 reduced proliferation but enhanced EGFR expression and cancer stem-like cells self-renewal enhancing undifferentiated state which could confer treatment resistance (Soeda et al., 2017). Furthermore, the predominant association among p38 MAPK activation (p-p38) with malignancy and progression in gliomas could be explained, in part, because high p38 or p-p38 levels are linked to increasing ROS, which is harmful to cancer cells and confers antitumor responses (Liu et al., 2015; Thiyagarajan et al., 2016; Liu et al., 2020).

### Clinical studies targeting p38 MAPK in cancer

Several clinical trials targeting p38 MAPK in cancer are currently underway. Ralimetinib (LY2228820), for example, is a potent and selective inhibitor of p38α and p38β and has been tested as a single agent and in combination with chemotherapeutic agents for the treatment of ovarian cancer, GBM, and metastatic breast cancer (Campbell et al., 2014; Vergote et al., 2020). An important clinical trial NCT02364206, including the ralimetinib, has been conducting to determine the recommended dose of the p38 inhibitor in combination with temozolomide and radiotherapy during chemoradiotherapy period (phase I) in patients with newly diagnosed GBM and to estimate the 6-month progression free survival (PFS) rate of patients when administered at the recommended dose in combination with radiotherapy and concomitant temozolomide (phase II).

Because of the wide-ranging controlling role of p38 in different cellular processes, the possibility of side effects resulting from an undesired pharmacological activity is a relevant concern for the p38 inhibitors. There are many discontinued p38 inhibitors that failed due to safety concerns, presenting adverse effects directed mostly to the hepatic system and skin (Xu et al., 2008; Karcher and Laufer, 2009; Yong et al., 2009). Other interesting approaches using CRISPR/Cas9 can allow the comparison of the altered gene expression profiles of the MAPK pathway members and the response to specific cancer treatments (Braicu et al., 2019). It is relevant to note that small molecules targeting p38 can cause a significant delay in cancer growth through multiple mechanisms, which becomes the development of p38 MAPK inhibitors with therapeutic benefits and reduced side effects a promising opportunity for future clinical studies to treat cancer.

## Conclusion

Malignant brain tumors are debilitating diseases with a dismal prognosis and extremely limited response to therapies. The aggressiveness of GBM has been marked by several signaling pathway dysregulations that are implicated in cell proliferation, survival, migration, and angiogenesis. Tumor cells are heterogeneous, so it is also important to note that targeting a mediator involved in multiple pathways would constitute a more efficient therapy. MAPKs are shown to support numerous processes of cell survival. The p38 MAPK pathway is a key route capable to influence different frameworks of cellular survival mechanisms such as inflammation, proliferation, migration, invasion, and ROS signaling. Briefly, the p38 MAPK is likely to be an oncogenic factor contributing to GBM initiation, progression, and chemotherapy resistance. Hence, a better understanding of the GBM tumor microenvironment is essential to the advancement of therapies that provide extended life expectancy for patients. Thus, targeting key components of these signaling pathways using small-molecule inhibitors, such as p38 inhibitors and antibodies, could provide progress in the treatment of gliomas.

## References

[B1] Anton D. B. Ducati R. G. Timmers L. F. S. M. Laufer S. Goettert M. I. (2021). A special view of what was almost forgotten: p38δ MAPK. Cancers (Basel) 2513 (9), 2077. 10.3390/cancers13092077 PMC8123357 33923030

[B2] Ardito F. Giuliani M. Perrone D. Troiano G. Lo Muzio L. (2017). The crucial role of protein phosphorylation in cell signaling and its use as targeted therapy (Review). Int. J. Mol. Med. 40, 271–280. 10.3892/ijmm.2017.3036 28656226 PMC5500920

[B3] Baier A. Szyszka R. (2020). Compounds from natural sources as protein kinase inhibitors. Biomolecules 10, 1546. 10.3390/biom10111546 PMC7698043 33198400

[B4] Berra E. Pagès G. Pouysségur J. (2000). MAP kinases and hypoxia in the control of VEGF expression. Cancer Metastasis Rev. 19, 139–145. 10.1023/a:1026506011458 11191053

[B5] Bononi A. Agnoletto C. De Marchi E. Marchi S. Patergnani S. Bonora M. (2011). Protein kinases and phosphatases in the control of cell fate. Enzyme Res. 2011, 329098. 10.4061/2011/329098 21904669 PMC3166778

[B6] Borst O. Walker B. Münzer P. Russo A. Schmid E. Faggio C. (2013). Skepinone-L, a novel potent and highly selective inhibitor of p38 MAP kinase, effectively impairs platelet activation and thrombus formation. Cell. Physiol. biochem. 31, 914–924. 10.1159/000350110 23817201

[B7] Braicu C. Buse M. Busuioc C. Drula R. Gulei D. Raduly L. (2019). A comprehensive review on MAPK: A promising therapeutic target in cancer. Cancers 11, E1618. 10.3390/cancers11101618 31652660 PMC6827047

[B8] Brancho D. Tanaka N. Jaeschke A. Ventura J. J. Kelkar N. Tanaka Y. (2003). Mechanism of p38 MAP kinase activation *in vivo* . Genes Dev. 17, 1969–1978. 10.1101/gad.1107303 12893778 PMC196252

[B9] Bulavin D. V. Fornace A. J. (2004). p38 MAP kinase’s emerging role as a tumor suppressor. Adv. Cancer Res. 92, 95–118. 10.1016/S0065-230X(04)92005-2 15530558

[B10] Campbell R. M. Anderson B. D. Brooks N. A. Brooks H. B. Chan E. M. De Dios A. (2014). Characterization of LY2228820 dimesylate, a potent and selective inhibitor of p38 MAPK with antitumor activity. Mol. Cancer Ther. 13, 364–374. 10.1158/1535-7163.MCT-13-0513 24356814

[B11] Cargnello M. Roux P. P. (2011). Activation and function of the MAPKs and their substrates, the MAPK-activated protein kinases. Microbiol. Mol. Biol. Rev. 75, 50–83. 10.1128/MMBR.00031-10 21372320 PMC3063353

[B12] Chaikuad A. Koch P. Laufer S. A. Knapp S. (2018). The cysteinome of protein kinases as a target in drug development. Angew. Chem. Int. Ed. Engl. 57, 4372–4385. 10.1002/anie.201707875 28994500

[B13] Chen X. Hao A. Li X. Ye K. Zhao C. Yang H. (2020). Activation of JNK and p38 MAPK mediated by ZDHHC17 drives glioblastoma multiforme development and malignant progression. Theranostics 10, 998–1015. 10.7150/thno.40076 31938047 PMC6956818

[B14] Chen Y. Gao F. Jiang R. Liu H. Hou J. Yi Y. (2017). Down-regulation of AQP4 expression via p38 MAPK signaling in temozolomide-induced glioma cells growth inhibition and invasion impairment. J. Cell. Biochem. 118, 4905–4913. 10.1002/jcb.26176 28569417

[B15] Choi C. Xu X. Oh J. W. Lee S. J. Gillespie G. Y. Park H. (2001). Fas-induced expression of chemokines in human glioma cells: Involvement of extracellular signal-regulated kinase 1/2 and p38 mitogen-activated protein kinase. Cancer Res. 61, 3084–3091. 11306491

[B16] Cohen P. (2002). The origins of protein phosphorylation. Nat. Cell Biol. 4, E127–E130. 10.1038/ncb0502-e127 11988757

[B17] Colquhoun A. (2017). Cell biology-metabolic crosstalk in glioma. Int. J. Biochem. Cell Biol. 89, 171–181. 10.1016/j.biocel.2017.05.022 28549626

[B18] Cuadrado A. Nebreda A. R. (2010). Mechanisms and functions of p38 MAPK signalling. Biochem. J. 429, 403–417. 10.1042/BJ20100323 20626350

[B19] Cuenda A. Rousseau S. (2007). p38 MAP-kinases pathway regulation, function and role in human diseases. Biochim. Biophys. Acta 1773, 1358–1375. 10.1016/j.bbamcr.2007.03.010 17481747

[B20] Cuenda A. Sanz-Ezquerro J. J. (2017). p38γ and p38δ: From spectators to key physiological players. Trends biochem. Sci. 42, 431–442. 10.1016/j.tibs.2017.02.008 28473179

[B21] Cuevas B. D. Abell A. N. Johnson G. L. (2007). Role of mitogen-activated protein kinase kinase kinases in signal integration. Oncogene 26, 3159–3171. 10.1038/sj.onc.1210409 17496913

[B22] Dang W. Xiao J. Ma Q. Miao J. Cao M. Chen L. (2021). Combination of p38 MAPK inhibitor with PD-L1 antibody effectively prolongs survivals of temozolomide-resistant glioma-bearing mice via reduction of infiltrating glioma-associated macrophages and PD-L1 expression on resident glioma-associated microglia. Brain Tumor Pathol. 38, 189–200. 10.1007/s10014-021-00404-3 34231121

[B23] Deacon K. Mistry P. Chernoff J. Blank J. L. Patel R. (2003). p38 Mitogen-activated protein kinase mediates cell death and p21-activated kinase mediates cell survival during chemotherapeutic drug-induced mitotic arrest. Mol. Biol. Cell 14, 2071–2087. 10.1091/mbc.e02-10-0653 12802076 PMC165098

[B24] DeAngelis L. M. (2001). Brain tumors. N. Engl. J. Med. 344, 114–123. 10.1056/NEJM200101113440207 11150363

[B25] Demuth T. Reavie L. B. Rennert J. L. Nakada M. Nakada S. Hoelzinger D. B. (2007). MAP-Ing glioma invasion: Mitogen-activated protein kinase kinase 3 and p38 drive glioma invasion and progression and predict patient survival. Mol. Cancer Ther. 6, 1212–1222. 10.1158/1535-7163.MCT-06-0711 17406030

[B26] Dhillon A. S. Hagan S. Rath O. Kolch W. (2007). MAP kinase signalling pathways in cancer. Oncogene 26, 3279–3290. 10.1038/sj.onc.1210421 17496922

[B27] Eder K. Kalman B. (2014). Molecular heterogeneity of glioblastoma and its clinical relevance. Pathol. Oncol. Res. 20, 777–787. 10.1007/s12253-014-9833-3 25156108

[B28] Gao C. F. Xie Q. Su Y. L. Koeman J. Khoo S. K. Gustafson M. (2005). Proliferation and invasion: Plasticity in tumor cells. Proc. Natl. Acad. Sci. U. S. A. 102, 10528–10533. 10.1073/pnas.0504367102 16024725 PMC1180792

[B29] García-Cano J. Roche O. Cimas F. J. Pascual-Serra R. Ortega-Muelas M. Fernández-Aroca D. M. (2016). p38MAPK and chemotherapy: We always need to hear both sides of the story. Front. Cell Dev. Biol. 4, 69. 10.3389/fcell.2016.00069 27446920 PMC4928511

[B30] Geest C. R. Coffer P. J. (2009). MAPK signaling pathways in the regulation of hematopoiesis. J. Leukoc. Biol. 86, 237–250. 10.1189/jlb.0209097 19498045

[B31] Glassmann A. Reichmann K. Scheffler B. Glas M. Veit N. Probstmeier R. (2011). Pharmacological targeting of the constitutively activated MEK/MAPK-dependent signaling pathway in glioma cells inhibits cell proliferation and migration. Int. J. Oncol. 39, 1567–1575. 10.3892/ijo.2011.1165 21850371

[B32] Goldsmith C. S. Kim S. M. Karunarathna N. Neuendorff N. Toussaint L. G. Earnest D. J. (2018). Inhibition of p38 MAPK activity leads to cell type-specific effects on the molecular circadian clock and time-dependent reduction of glioma cell invasiveness. BMC Cancer 18, 43. 10.1186/s12885-017-3896-y 29316898 PMC5761097

[B33] Grossi V. Peserico A. Tezil T. Simone C. (2014). p38α MAPK pathway: A key factor in colorectal cancer therapy and chemoresistance. World J. Gastroenterol. 20, 9744–9758. 10.3748/wjg.v20.i29.9744 25110412 PMC4123363

[B34] Haller V. Nahidino P. Forster M. Laufer S. A. (2020). An updated patent review of p38 MAP kinase inhibitors (2014-2019). Expert Opin. Ther. Pat. 30, 453–466. 10.1080/13543776.2020.1749263 32228113

[B35] Han J. Lee J. D. Bibbs L. Ulevitch R. J. (1994). A MAP kinase targeted by endotoxin and hyperosmolarity in mammalian cells. Science 265, 808–811. 10.1126/science.7914033 7914033

[B36] Han L. Yuan B. Shimada R. Hayashi H. Si N. Zhao H. Y. (2018). Cytocidal effects of arenobufagin and hellebrigenin, two active bufadienolide compounds, against human glioblastoma cell line U-87. Int. J. Oncol. 53, 2488–2502. 10.3892/ijo.2018.4567 30272276 PMC6203163

[B37] Hanahan D. Weinberg R. A. (2011). Hallmarks of cancer: The next generation. Cell 144, 646–674. 10.1016/j.cell.2011.02.013 21376230

[B38] Hanif F. Muzaffar K. Perveen K. Malhi S. M. Simjee S. U. (2017). Glioblastoma multiforme: A review of its epidemiology and pathogenesis through clinical presentation and treatment. Asian pac. J. Cancer Prev. 18, 3–9. 10.22034/APJCP.2017.18.1.3 28239999 PMC5563115

[B39] Johnson G. L. Dohlman H. G. Graves L. M. (2005). MAPK kinase kinases (MKKKs) as a target class for small-molecule inhibition to modulate signaling networks and gene expression. Curr. Opin. Chem. Biol. 9, 325–331. 10.1016/j.cbpa.2005.04.004 15939336

[B40] Jones D. T. W. Gronych J. Lichter P. Witt O. Pfister S. M. (2012). MAPK pathway activation in pilocytic astrocytoma. Cell. Mol. Life Sci. 69, 1799–1811. 10.1007/s00018-011-0898-9 22159586 PMC3350769

[B41] Karcher S. C. Laufer S. A. (2009). Successful structure-based design of recent p38 MAP kinase inhibitors. Curr. Top. Med. Chem. 9 (7), 655–676. 10.2174/156802609789007363 19689372

[B42] Kelly G. Strasser A. (2011). The essential role of evasion from cell death in cancer. Adv. Cancer Res. 111, 39–96. 10.1016/B978-0-12-385524-4.00002-7 21704830 PMC3128425

[B43] Keshet Y. Seger R. (2010). The MAP kinase signaling cascades: A system of hundreds of components regulates a diverse array of physiological functions. Methods Mol. Biol. 661, 3–38. 10.1007/978-1-60761-795-2_1 20811974

[B44] Kim E. K. Choi E. J. (2015). Compromised MAPK signaling in human diseases: An update. Arch. Toxicol. 89, 867–882. 10.1007/s00204-015-1472-2 25690731

[B45] Koul H. K. Pal M. Koul S. (2013). Role of p38 MAP kinase signal transduction in solid tumors. Genes Cancer 4, 342–359. 10.1177/1947601913507951 24349632 PMC3863344

[B46] Kühnöl C. Herbarth M. Föll J. Staege M. S. Kramm C. (2013). CD137 stimulation and p38 MAPK inhibition improve reactivity in an *in vitro* model of glioblastoma immunotherapy. Cancer Immunol. Immunother. 62, 1797–1809. 10.1007/s00262-013-1484-9 24129764 PMC11028552

[B47] Lapointe S. Perry A. Butowski N. A. (2018). Primary brain tumours in adults. Lancet 392, 432–446. 10.1016/S0140-6736(18)30990-5 30060998

[B48] Lee S. Rauch J. Kolch W. (2020). Targeting MAPK signaling in cancer: Mechanisms of drug resistance and sensitivity. Int. J. Mol. Sci. 21, E1102. 10.3390/ijms21031102 32046099 PMC7037308

[B49] Lei Y. Y. Wang W. J. Mei J. H. Wang C. L. (2014). Mitogen-activated protein kinase signal transduction in solid tumors. Asian pac. J. Cancer Prev. 15, 8539–8548. 10.7314/apjcp.2014.15.20.8539 25374165

[B50] Li H. Liu Y. Gu Z. Li L. Liu Y. Wang L. (2018). p38 MAPK-MK2 pathway regulates the heat-stress-induced accumulation of reactive oxygen species that mediates apoptotic cell death in glial cells. Oncol. Lett. 15, 775–782. 10.3892/ol.2017.7360 29387240 PMC5768138

[B51] Lin L. Cai J. Jiang C. (2017). Recent advances in targeted therapy for glioma. Curr. Med. Chem. 24, 1365–1381. 10.2174/0929867323666161223150242 28019637

[B52] Liu Q. Zou R. Zhou R. Gong C. Wang Z. Cai T. (2015). miR-155 regulates glioma cells invasion and chemosensitivity by p38 isforms *in vitro* . J. Cell. Biochem. 116, 1213–1221. 10.1002/jcb.25073 25535908

[B53] Liu W. Chai Y. Hu L. Wang J. Pan X. Yuan H. (2020). Polyphyllin VI induces apoptosis and autophagy via reactive oxygen species mediated JNK and P38 activation in glioma. Onco. Targets. Ther. 13, 2275–2288. 10.2147/OTT.S243953 32214827 PMC7078907

[B54] Loesch M. Chen G. (2008). The p38 MAPK stress pathway as a tumor suppressor or more? Front. Biosci. 13, 3581–3593. 10.2741/2951 18508457 PMC4758212

[B55] Ma J. Su L. J. Zheng Z. J. (2018). Upregulation of long noncoding RNA MRCCAT1 predicts poor prognosis and functions as an oncogene in glioma. Eur. Rev. Med. Pharmacol. Sci. 22, 8406–8414. 10.26355/eurrev_201812_16539 30556882

[B56] Ma L. Liu J. Zhang X. Qi J. Yu W. Gu Y. (2015). p38 MAPK-dependent Nrf2 induction enhances the resistance of glioma cells against TMZ. Med. Oncol. 32, 69. 10.1007/s12032-015-0517-y 25691294

[B57] Mai L. Zhu X. Huang F. He H. Fan W. (2020). p38 mitogen-activated protein kinase and pain. Life Sci. 256, 117885. 10.1016/j.lfs.2020.117885 32485175

[B58] Martínez-Limón A. Joaquin M. Caballero M. Posas F. de Nadal E. (2020). The p38 pathway: From biology to cancer therapy. Int. J. Mol. Sci. 21 (6), 1913. 10.3390/ijms21061913 PMC7139330 32168915

[B59] Mohan A. A. Tomaszewski W. H. Haskell-Mendoza A. P. Hotchkiss K. M. Singh K. Reedy J. L. (2021). Targeting immunometabolism in glioblastoma. Front. Oncol. 11, 696402. 10.3389/fonc.2021.696402 34222022 PMC8242259

[B60] Morrone F. B. Gehring M. P. Nicoletti N. F. (2016). Calcium channels and associated receptors in malignant brain tumor therapy. Mol. Pharmacol. 90, 403–409. 10.1124/mol.116.103770 27418672

[B61] Morrone F. B. Vargas P. Rockenbach L. Scheffel T. B. (2021). P2Y12 purinergic receptor and brain tumors: Implications on glioma microenvironment. Molecules 26, 6146. 10.3390/molecules26206146 34684726 PMC8540665

[B62] Niu H. Li X. Yang A. Jin Z. Wang X. Wang Q. (2018). Cycloartenol exerts anti-proliferative effects on Glioma U87 cells via induction of cell cycle arrest and p38 MAPK-mediated apoptosis. J. BUON. 23, 1840–1845. 30610811

[B63] Nørøxe D. S. Poulsen H. S. Lassen U. (2017). Hallmarks of glioblastoma: A systematic review. ESMO Open 1, e000144. 10.1136/esmoopen-2016-000144 28912963 PMC5419216

[B64] O’Callaghan C. Fanning L. J. Barry O. P. (2015). p38δ MAPK phenotype: an indicator of chemotherapeutic response in oesophageal squamous cell carcinoma. Anticancer. Drugs 26, 46–55. 10.1097/CAD.0000000000000156 25099621 PMC4243785

[B65] Oliver L. Olivier C. Marhuenda F. B. Campone M. Vallette F. M. (2009). Hypoxia and the malignant glioma microenvironment: Regulation and implications for therapy. Curr. Mol. Pharmacol. 2, 263–284. 10.2174/1874467210902030263 20021464

[B66] Olson J. M. Hallahan A. R. (2004). p38 MAP kinase: a convergence point in cancer therapy. Trends Mol. Med. 10, 125–129. 10.1016/j.molmed.2004.01.007 15102355

[B67] Omuro A. DeAngelis L. M. (2013). Glioblastoma and other malignant gliomas: A clinical review. JAMA 310, 1842–1850. 10.1001/jama.2013.280319 24193082

[B68] Ono K. Han J. (2000). The p38 signal transduction pathway: Activation and function. Cell. Signal. 12, 1–13. 10.1016/s0898-6568(99)00071-6 10676842

[B69] Ostrom Q. T. Gittleman H. Truitt G. Boscia A. Kruchko C. Barnholtz-Sloan J. S. (2018). CBTRUS statistical report: Primary brain and other central nervous system tumors diagnosed in the United States in 2011-2015. Neuro. Oncol. 20, iv1. 10.1093/neuonc/noy131 30445539 PMC6129949

[B70] Ott P. A. Adams S. (2011). Small-molecule protein kinase inhibitors and their effects on the immune system: Implications for cancer treatment. Immunotherapy 3 (2), 213–227. 10.2217/imt.10.99 Feb 21322760 PMC4009988

[B71] Pandey V. Bhaskara V. K. Babu P. P. (2016). Implications of mitogen-activated protein kinase signaling in glioma. J. Neurosci. Res. 94, 114–127. 10.1002/jnr.23687 26509338

[B72] Park C. M. Park M. J. Kwak H. J. Lee H. C. Kim M. S. Lee S. H. (2006). Ionizing radiation enhances matrix metalloproteinase-2 secretion and invasion of glioma cells through Src/epidermal growth factor receptor-mediated p38/Akt and phosphatidylinositol 3-kinase/Akt signaling pathways. Cancer Res. 66, 8511–8519. 10.1158/0008-5472.CAN-05-4340 16951163

[B73] Patil C. G. Nuño M. Elramsisy A. Mukherjee D. Carico C. Dantis J. (2013). High levels of phosphorylated MAP kinase are associated with poor survival among patients with glioblastoma during the temozolomide era. Neuro. Oncol. 15, 104–111. 10.1093/neuonc/nos272 23115159 PMC3534422

[B74] Pearson G. Robinson F. Beers Gibson T. Xu B. E. Karandikar M. Berman K. (2001). Mitogen-activated protein (MAP) kinase pathways: Regulation and physiological functions. Endocr. Rev. 22, 153–183. 10.1210/edrv.22.2.0428 11294822

[B75] Peluso I. Yarla N. S. Ambra R. Pastore G. Perry G. (2019). MAPK signalling pathway in cancers: Olive products as cancer preventive and therapeutic agents. Semin. Cancer Biol. 56, 185–195. 10.1016/j.semcancer.2017.09.002 28912082

[B76] Perry A. Wesseling P. (2016). Histologic classification of gliomas. Handb. Clin. Neurol. 134, 71–95. 10.1016/B978-0-12-802997-8.00005-0 26948349

[B77] Pojo M. Marques B. (2011). “Molecular hallmarks of gliomas,” in Mol. Targets CNS tumors. Editor Garami M. (London: InTech). 10.5772/21352

[B78] Reni M. Mazza E. Zanon S. Gatta G. Vecht C. J. (2017). Central nervous system gliomas. Crit. Rev. Oncol. Hematol. 113, 213–234. 10.1016/j.critrevonc.2017.03.021 28427510

[B79] Reyskens K. M. S. E. Arthur J. S. C. (2016). Emerging roles of the mitogen and stress activated kinases MSK1 and MSK2. Front. Cell Dev. Biol. 4, 56. 10.3389/fcell.2016.00056 27376065 PMC4901046

[B80] Roche O. Fernández-Aroca D. M. Arconada-Luque E. García-Flores N. Mellor L. F. Ruiz-Hidalgo M. J. (2020). p38β and cancer: The beginning of the road. Int. J. Mol. Sci. 21, 7524. 10.3390/ijms21207524 PMC7589630 33053909

[B81] Rodríguez-García A. Samsó P. Fontova P. Simon-Molas H. Manzano A. Castaño E. (2017). TGF-β1 targets Smad, p38 MAPK, and PI3K/Akt signaling pathways to induce PFKFB3 gene expression and glycolysis in glioblastoma cells. FEBS J. 284, 3437–3454. 10.1111/febs.14201 28834297

[B82] Scheffel T. B. Grave N. Vargas P. Diz F. M. Rockenbach L. Morrone F. B. (2021). Immunosuppression in gliomas via PD-1/PD-L1 Axis and adenosine pathway. Front. Oncol. 10, 617385. 10.3389/fonc.2020.617385 33659213 PMC7919594

[B83] Schieven G. L. (2009). The p38alpha kinase plays a central role in inflammation. Curr. Top. Med. Chem. 9, 1038–1048. 10.2174/156802609789630974 19747121

[B84] Schiffer D. Annovazzi L. Casalone C. Corona C. Mellai M. (2018). Glioblastoma: Microenvironment and niche concept. Cancers 11, 5. 10.3390/cancers11010005 PMC6357107 30577488

[B85] Sebolt-Leopold J. S. Herrera R. (2004). Targeting the mitogen-activated protein kinase cascade to treat cancer. Nat. Rev. Cancer 4, 937–947. 10.1038/nrc1503 15573115

[B86] Soeda A. Lathia J. Williams B. J. Wu Q. Gallagher J. Androutsellis-Theotokis A. (2017). The p38 signaling pathway mediates quiescence of glioma stem cells by regulating epidermal growth factor receptor trafficking. Oncotarget 8, 33316–33328. 10.18632/oncotarget.16741 28410196 PMC5464870

[B87] Sooman L. Lennartsson J. Gullbo J. Bergqvist M. Tsakonas G. Johansson F. (2013). Vandetanib combined with a p38 MAPK inhibitor synergistically reduces glioblastoma cell survival. Med. Oncol. 30, 638. 10.1007/s12032-013-0638-0 23783486

[B88] Stramucci L. Pranteda A. Bossi G. (2018). Insights of crosstalk between p53 protein and the MKK3/MKK6/p38 MAPK signaling pathway in cancer. Cancers 10, E131. 10.3390/cancers10050131 29751559 PMC5977104

[B89] Tang F. Wang H. Chen E. Bian E. Xu Y. Ji X. (2019). LncRNA-ATB promotes TGF-β-induced glioma cells invasion through NF-κB and P38/MAPK pathway. J. Cell. Physiol. 234, 23302–23314. 10.1002/jcp.28898 31140621

[B90] Tate C. M. Blosser W. Wyss L. Evans G. Xue Q. Pan Y. (2013). LY2228820 dimesylate, a selective inhibitor of p38 mitogen-activated protein kinase, reduces angiogenic endothelial cord formation *in vitro* and *in vivo* . J. Biol. Chem. 288, 6743–6753. 10.1074/jbc.M112.425553 23335506 PMC3585111

[B91] Thiyagarajan V. Sivalingam K. S. Viswanadha V. P. Weng C. F. (2016). 16-hydroxy-cleroda-3, 13-dien-16, 15-olide induced glioma cell autophagy via ROS generation and activation of p38 MAPK and ERK-1/2. Environ. Toxicol. Pharmacol. 45, 202–211. 10.1016/j.etap.2016.06.005 27318969

[B92] Vergote I. Heitz F. Buderath P. Powell M. Sehouli J. Lee C. M. (2020). A randomized, double-blind, placebo-controlled phase 1b/2 study of ralimetinib, a p38 MAPK inhibitor, plus gemcitabine and carboplatin versus gemcitabine and carboplatin for women with recurrent platinum-sensitive ovarian cancer. Gynecol. Oncol. 156, 23–31. 10.1016/j.ygyno.2019.11.006 31791552

[B93] Wada T. Penninger J. M. (2004). Mitogen-activated protein kinases in apoptosis regulation. Oncogene 23, 2838–2849. 10.1038/sj.onc.1207556 15077147

[B94] Wagner E. F. Nebreda Á. R. (2009). Signal integration by JNK and p38 MAPK pathways in cancer development. Nat. Rev. Cancer 9, 537–549. 10.1038/nrc2694 19629069

[B95] Wang H. Guan Q. Nan Y. Ma Q. Zhong Y. (2019). Overexpression of human MX2 gene suppresses cell proliferation, migration, and invasion via ERK/P38/NF-κB pathway in glioblastoma cells. J. Cell. Biochem. 120, 18762–18770. 10.1002/jcb.29189 31265172

[B96] Wittlinger F. Laufer S. A. (2021). The pre-clinical discovery and development of osimertinib used to treat non-small cell lung cancer. Expert Opin. Drug Discov. 16, 1091–1103. 10.1080/17460441.2021.1936496 34053372

[B97] Xu J. J. Hendriks B. S. Zhao J. Graaf D. (2008). Multiple effects of acetaminophen and p38 inhibitors: Towards pathway toxicology. FEBS Lett. 582 (8), 1276–1282. 10.1016/j.febslet.2008.01.063 18282474

[B98] Xu Y. Sun Q. Yuan F. Dong H. Zhang H. Geng R. (2020). RND2 attenuates apoptosis and autophagy in glioblastoma cells by targeting the p38 MAPK signalling pathway. J. Exp. Clin. Cancer Res. 39, 174. 10.1186/s13046-020-01671-2 32867814 PMC7457501

[B99] Yang K. Liu Y. Liu Z. Liu J. Liu X. Chen X. (2013). p38γ overexpression in gliomas and its role in proliferation and apoptosis. Sci. Rep. 3, 2089. 10.1038/srep02089 23807566 PMC3695572

[B100] Yeung Y. T. Bryce N. S. Adams S. Braidy N. Konayagi M. McDonald K. L. (2012). p38 MAPK inhibitors attenuate pro-inflammatory cytokine production and the invasiveness of human U251 glioblastoma cells. J. Neurooncol. 109, 35–44. 10.1007/s11060-012-0875-7 22528800

[B101] Yeung Y. T. McDonald K. L. Grewal T. Munoz L. (2013). Interleukins in glioblastoma pathophysiology: Implications for therapy. Br. J. Pharmacol. 168, 591–606. 10.1111/bph.12008 23062197 PMC3579281

[B102] Yong H. Y. Koh M. S. Moon A. (2009). The p38 MAPK inhibitors for the treatment of inflammatory diseases and cancer. Expert Opin. Investig. Drugs 18 (12), 1893–1905. 10.1517/13543780903321490 19852565

[B103] Yoshino Y. Aoyagi M. Tamaki M. Duan L. Morimoto T. Ohno K. (2006). Activation of p38 MAPK and/or JNK contributes to increased levels of VEGF secretion in human malignant glioma cells. Int. J. Oncol. 29, 981–987. 10.3892/ijo.29.4.981 16964394

[B104] Yue J. López J. M. (2020). Understanding MAPK signaling pathways in apoptosis. Int. J. Mol. Sci. 21, 2346. 10.3390/ijms21072346 PMC7177758 32231094

[B105] Zhang J. Shen B. Lin A. (2007). Novel strategies for inhibition of the p38 MAPK pathway. Trends Pharmacol. Sci. 28, 286–295. 10.1016/j.tips.2007.04.008 17482683

[B106] Zhao L. Wang Y. Xu Y. Sun Q. Liu H. Chen Q. (2021). BIRB796, an inhibitor of p38 mitogen-activated protein kinase, inhibits proliferation and invasion in glioblastoma cells. ACS Omega 6, 11466–11473. 10.1021/acsomega.1c00521 34056302 PMC8154025

[B107] Zhou Y. Chu X. Deng F. Tong L. Tong G. Yi Y. (2017). The adenosine A2b receptor promotes tumor progression of bladder urothelial carcinoma by enhancing MAPK signaling pathway. Oncotarget 8, 48755–48768. 10.18632/oncotarget.17835 28548944 PMC5564722

[B108] Zou X. Blank M. (2017). Targeting p38 MAP kinase signaling in cancer through post-translational modifications. Cancer Lett. 384, 19–26. 10.1016/j.canlet.2016.10.008 27725227

